# Reparation Policy in Gendered Political Violence: Gendered Torture During the Dictatorship and the Recent “Social Explosion” in Chile

**DOI:** 10.1007/s41134-023-00248-1

**Published:** 2023-05-22

**Authors:** Carla Cubillos-Vega

**Affiliations:** Departamento de Trabajo Social y Servicios Sociales, Universidad Complutense de Madrid, Madrid, Spain

**Keywords:** Reparation policies, Non-repetition assurances, Sexual violence, Gendered torture, Human rights

## Abstract

This study explores the gendered violence dimension present in the torture exerted in Chile and the problems that continue to affect the reparation policies. The analysis covers the cases of political prisoners during the Chilean dictatorship (1973–1990) and that of the people detained within the context of the social protest of October 18, 2019. The methodology used for this study includes desk research on secondary sources on gendered political violence and torture such as scholarly books, journalistic and academic articles, and non-governmental organization reports, analyzing their contents from a perspective based on human rights and gender. We argue that the crystallization of gender-based violence exerted by Chilean State agents is linked to the biases present in post-dictatorship reparation policy and reflect on the impact of these biases on the assurances of non-repetition of human rights violations.

## Introduction

Within the sphere of international human rights law, victims of human rights violations have been granted a number of rights: to truth, justice, and reparation, as well as to the implementation of assurances of non-repetition (Joinet, 1997). These rights coincide (although not exclusively) with the field of the so-called transitional justice (TJ), which deals with human rights violations of the recent past, which have taken place within the context of armed conflicts and/or political transitions. Regarding the realization of these rights, reparation measures can take the form of restitution, indemnification, rehabilitation, satisfaction, and assurances of non-repetition (Van Boven, 1993; UN, 2005). The right to reparation, in a broad sense, involves a compensation for the damage suffered (reparation as such) and the resumption of the rule of law.

In Chile, the armed forces led a coup d’état in 1973 that overthrew the democratic socialist government of President Salvador Allende, establishing a civic-military dictatorship led by General Augusto Pinochet (1973–1990). For 17 years, Pinochet’s Regime committed systematic human rights violations to exterminate their opponents. When the dictatorship ended in 1990, reparation policies implemented in response to human rights violations were derived from the recommendations of the National Truth and Reconciliation Commission, published on the Rettig Report (1991). Said policies have been insufficient, since they have failed to yield the whole truth and actual justice, have excluded the experiences of violence of its sufferers, and have been circumscribed to specific sectors (Bustamante-Danilo & Carreño-Calderón, 2020; Collins, 2019; Hiner, 2009; Jara et al., 2018; Lira, 1996, 2011; Lira & Loveman, 2005). Furthermore, tortured political prisoners were the last ones to benefit from reparation policies. Only in 2003, in order to meet their demands, a second Truth Commission was created as a way of identifying the people who had been imprisoned and tortured for political reasons during the dictatorship. The fruit of this commission’s efforts was the Valech Report, published in 2005, which acknowledged nearly 30 thousand victims, identified 1132 detention and torture centers, and made a number of reparation recommendations (Hopenhayn, 2018; Hourcade et al., 2018). Nevertheless, the overwhelming presence of gendered violence in these cases and the impunity surrounding them have remained invisible (Tito, 2019). Within the more recent context of the state of emergency and curfews after the wave of social protests that began on October 18, 2019—the so-called social explosion (18-O)—order and security forces have violently and systematically repressed demonstrators. Against this backdrop, reports have been made of the existence of torture centers akin to those used during the dictatorship. Cases of sexual abuse and rape have been documented at clandestine centers as well as public detention places and police stations (Bermúdez, 2019; INDH, 2019).

Considering the above, the aim of this study is to analyze the gendered violence dimension present both in the torture exerted in Chile during the dictatorship and within the context of the recent wave of protests or 18-O. Both events are considered part of a continuum of political violence that did not finish at the end of the dictatorship. In this line, we explore the biases found in victim reparation policies and non-repetition assurances, which have persisted to this day. Specifically, the study focuses on the institutional political violence exerted in the form of sexual violence and torture. Although this topic lies partly within the sphere of TJ, it exceeds it because it also covers the recent situation of the 18-O victims. Yet, the latter case clearly illustrates the failure of the assurances of non-repetition that should have been implemented after the start of the return to democracy in Chile.

The study’s relevance lies in examining issues that remain largely unexplored from the perspective described above. Furthermore, the topic addressed is relevant because of its crucial social presence, given that political violence—at the time of writing this article—has not yet ceased.

On the other hand, this work contributes to social work since our profession, besides being a human rights profession (Healy, 2008; Ife, 2001; Cubillos-Vega, 2019), also has an essential role in conflict resolution and recovery from violence (Androff, 2010). Participation of social workers in the teams that design and implement transitional justice measures in the form of reparation (truth and reconciliation commissions or health reparation programs) is frequent (Androff, 2010;  Cubillos Vega, 2023).

As Truth and Reconciliation Commissions are considered a reparation measure, the intervention of social workers on these contexts is a reparative work. Androff (2010) shows some examples of the manners social workers have engaged with Truth and Reconciliation Commissions in the past. In South Africa, social workers delivered direct services to the victims (as counselling and therapeutic support services) and contributed with policy making and satisfaction measures as they “engaged in professional advocacy by submitting official statements to the TRC denouncing their previous support for Apartheid policies of discrimination and oppression” (p. 1963).

Formal apologies given by state agents are one of the most common reparation measures in transitional justice contexts. Likewise, on policy-making actions, based on the investigations and documentation of past abuses collected in the truth and reconciliation commissions (TRCs) in Timor Leste, Australian social workers participated in the design of a mental health service delivery system to meet the needs of Timorese victims suffering from psychological disorders (Androff, 2010).

TRCs are not just fields where social workers can provide their expertise in reparations for human rights violations. In the Conosur, Argentinian, Brazilian, Chilean, and Uruguayan social workers had to struggle with the dictatorships. As Eroles (1997) states, in human right violation contexts, social work has been a risk profession since ethical and politics commitment is usually a reason for professional persecution. Even so, in Argentina and Chile, social workers accompanied victims during the dictatorship, providing social assistance to political prisoners and their families, promoting the social insertion of released persons, and providing support, guidance, and legal advice to the families of disappeared persons (Gil de Camín, 1997). Later, after the end of the dictatorship, they actively participated in creating reparation policies (such as truth and reparation commissions) and providing services to repair the physical and moral damage suffered. An example of these policies would be the social worker’s intervention in the Programme of Assistance to Victims of State Terrorism in Argentina, accompanying victims in trials for crimes against humanity (Ardohain, 2014). In Chile, social workers participate in the Comprehensive Health Care and Reparation Programme (PRAIS). PRAIS was ethically and legally based on the recommendations of the TRC called Comisión Nacional de Verdad y Reconciliación (CNVR) and was designed to provide people affected by the political repression of the State with specialized physical and mental health care throughout the country (Hayner, 2001; Castañeda & Salame, 2019; Cubillos Vega, 2023). In this sense, the case of Chile is paradigmatic, as it was the first country to implement a Reparation and Integral Health Care Program (PRAIS) where social work has been present, since 1990, in the multidisciplinary teams that have designed and implemented both the CNVR and the PRAIS program.

## Methodology and Materials

Using a qualitative approach, this analysis adopted the concept of gender-based torture. We argue that gender-based violence exerted by Chilean State agents during the dictatorship has been crystallized and its recent rise is linked to the biases present in the reparation policy after the dictatorship. Within the framework of this approach, we sought out and extracted evidence from various sources of documentation on gendered political violence and torture: archives, scholarly books, journalistic and academic articles, and NGO reports. The data collected were analyzed in order to comprehend and interpret, adopting an inductive approach (Denzin & Lincoln, 1994) from a perspective based on human rights and gender. The study covers two cases that, as we state in the introduction, illustrate the continuity of political gender-based violence: (a) women imprisoned and tortured for political reasons during the Chilean dictatorship (1973–1990) and (b) the people detained within the context of the recent wave of protests that began on October 18, 2019, also known as the “social explosion” or 18-O, where violence became more frequent.

## Sexual Violence and Gender-Based Torture

Gender-based violence within the context of crimes against humanity in Latin American armed conflicts has been mostly studied in connection with the cases of Colombia, Guatemala, and Argentina, with the latter being profusely researched. The invisibilization of the experience of women victims of sexual violence and the absence of a suitable reparation policy are among the most common findings of studies on gender-based violence—in the form of State violence (institutional political violence)—in Latin America, particularly in Southern Cone countries. In Central America, the armed conflict in Guatemala is a clear example of the use of sexual violence as a specific form of torture directed at women (especially those of the Maya people), through both individual and selective sexual violence as well as mass, public rapes (González, 2014). With respect to the Colombian armed conflict, it is relevant to note the jurisprudence of the country’s Supreme Court (Camargo et al., 2011), although authors have criticized the exclusion of violence against women from the sphere of TJ, a fact that reflects the gender inequalities and discrimination that underlie Colombian society (Lyons, 2011). The same criticism has been leveled in Argentina (Bilbao, 2011; Tornay & Alvarez, 2012; Cerruti, 2017; Olivera-Williams, 2017), since TJ policies established a notion of justice that rendered invisible the situation of women victims of sexual violence due to its focus on the figure of the disappeared, thus overlooking historically entrenched gender oppression.

In Chile, the situation has been similar, but gender-based torture experiences are less present in the scientific literature. As Hiner points out, few academic texts directly examine the topic of sexual torture against women political prisoners during the Chilean dictatorship, largely because few women have dared to reveal their experience (Hiner, 2015). More recent studies also emphasize, along with the invisibilization of women’s situation (Bunster, 1991; Hiner, 2009; Maravall, 2008) and sexual violence as torture (Jamett, 2012; Palestro, 2012), how said sexual and gender-based violence punished political as well as sexual and gender deviations in an essentially patriarchal society (Maravall, 2008; Olivera-Williams, 2017). Hiner’s study (2009) is a relevant reference point for the present study since the author reviews—from a gender-informed perspective—two of Chile’s most relevant TJ policies: the Rettig Report (1991) and the Valech Report (2005), analyzing how these documents constructed a hegemonic, stereotyped, and binary victim-perpetrator category that excluded certain experiences of women as well as their agency while also marginalizing other constructions of the official narrative and promoting a divide between past and present experiences of gender-based violence.

Buckley-Zistel et al. (2014) stress that feminist theories play a key role in illustrating how the field of TJ is thoroughly influenced by gender; nevertheless, this field has only partially addressed women’s experience in Latin American contexts. Gender violence, a key concept developed by feminist theorists, encompasses the sexual violence typical of gender-based torture. As noted by Segato (2003), sexual violence is always gender-based, even when exerted against people other than women, since it is defined by the use and abuse of the other’s body based on a power structure where the target is the female signifier—subjected, weak, conquered, or dominated—in order to crush people’s will and agency through an attack on their bodies. This mechanism of submission has been identified by Elizabeth Odio, who considers that the rape of women in armed conflicts constitutes a systematic policy adopted to destroy both: groups and direct victims (Odio, 1997). Segato (2020) notes that, from the rapist’s point of view, rape is pedagogical, based on the assumption that it helps to bind the victim to the patriarchal order. Thus, the main difference between the rapes that take place within personal relationships and those affecting generic victims who occupy a specific social or political category is that, in the latter case, rape constitutes a weapon of war. To encompass all the manifestations of this phenomenon, the author proposes the term *femigenocide*, a concept that alludes to a generic, impersonal, and systematic crime aimed at destructing large numbers of victims—both men and women—due to their feminized status (Segato, 2016, 2020).

In the field of torture studies, authors have developed three increasingly complex concepts: sexualized torture, gender-based torture, and gendered torture (Pérez-Sales & Zraly, 2018). The term *sexualized torture* refers to any act—either verbal, emotional, or physical—associated with sexuality which is performed in order to cause a person physical or psychological suffering. The notion of gender-based torture is centered on the gender identity of the actors; that is, it rests on the assumption that, even if an act of torture is not of a sexual nature, its form will be based on the gender identity of the person tortured. Lastly, the concept of gendered torture expands on the latter, emphasizing *that which is “gendered”* in gender-based torture. This term focuses on the following: (1) the influence of gender on the meaning and purpose of torture, (2) the role of torture in the preservation of gender hierarchies, and (3) the association between torture and experience, embodiment, and the performance of gender and other features (considering intersectionality).

These three definitions are not mutually exclusive: an act of sexual violence constitutive of torture (e.g., rape) integrates all three dimension or conceptions, depending on the analytic complexity applied. Nevertheless, for a policy on torture to have a critical gender approach, it should adopt the definition of gender-based or gendered torture. According to the authors, gender-aware analyses of torture must incorporate a wide range of human rights violations, from rape and attacks on sexual integrity to any other inflicted suffering closely associated with gender, including—but not limited to—sexual and identity dissidences (Pérez-Sales & Zraly, 2018). This need for reconceptualization also entails the need to reformulate the mechanisms for detecting and evaluating torture (such as the Istanbul Protocol) in order to integrate a feminist gender perspective into reparation policies (Pérez-Sales & Zraly, 2018).

## Gender-Based Torture in Chile During the Civilian-Military Dictatorship

First, it is worth noting that, in all Southern Cone dictatorships, female sexual slavery, historically employed by the State in a generic and diffuse manner, became materialized through the military State as a torturer (Bunster, 1991; Hiner, 2009). The torture of women political prisoners in Southern Cone dictatorships (in Argentina, Chile, and Uruguay), exerted as a form of sexual slavery, exhibits a dual pattern found in its physical and psychological manifestations: (a) using prisoners as an instrument of psychological torture aimed at “their” men, in order to punish them for their political activism; and (b) deliberately brutalizing and extinguishing them as human beings, since they were women “whose political consciousness drove them to political activism as a way of establishing a fairer social order in their countries” (Bunster, 1991, p. 47). As the author points out, this was the case of Chilean women who, during Allende’s regime, worked toward creating a more egalitarian socioeconomic order and then strove to liberate the country from the dictatorship. During the dictatorship, both men and women were tortured with rather similar methods. However, Bunster (1991) stresses that specific techniques were designed considering women’s sexual identity and anatomy. Apart from brutal damage to their reproductive organs (e.g., electricity, burns, beatings, cuts), sexual slavery was among the most prevalent torture methods, with sexual violence playing a key control role in interrogations and punishment.

Among the most frequent acts of sexual violence geared toward social control, rape (with animals, with objects, by groups of people, in front of the prisoners’ families, among other brutal varieties) was a form of “instruction,” as defined by Segato (2020), aimed at compelling victims to respect their traditional roles in society: mother, wife, a man’s partner—a socially inferior, secondary role. The moral and physical humiliation and degradation that these rapes caused were also intended to destroy the victims socially, culturally, and politically by undermining their social respect, grounded in their traditional roles, as well as their own self-respect (Bunster, 1991). Lastly, we should not overlook the racial and class components of gendered torture: the intersectionality of being a woman, from a proletarian background, and with mixed race or native features, increased the likelihood of more severe sexual torture, in contrast with the case of white women from bourgeois families (Bunster, 1991).

In terms of figures, according to the Report of the National Commission on Political Imprisonment and Torture (CNPPT), of the 35,868 testimonies received by the Commission, 27,255 people were classed as victims of political imprisonment and torture, 3399 of whom were women (12.5%) (CNPPT, 2005). Of these women, 316 declared having been raped (though to a lesser extent, the Report also mentions men who were raped). Nevertheless, since for many women sexual violence is difficult to verbalize and share, rape is believed to be underrepresented in the official data (Lira, 2017). Of them, 229 women were detained and tortured while pregnant, and some of them gave birth in captivity (Bunster, 1991; CNPPT, 2005; Hopenhayn, 2018; Lira, 2017; Palestro, 2012).

## Gender Biases in Torture Reparation Policies

Chilean lawmakers have produced two relevant TJ policies, which have defined an official truth and laid the groundwork (a conceptual and normative framework) for the design of subsequent measures, derived from the suggestions of the Rettig (1991) and Valech Reports (2005). In any case, people imprisoned and tortured for political reasons were not considered in the initial reparation policy and were only acknowledged and classified by the CNPPT one decade after the return to democracy.

As previously noted, public international law has progressively recognized that sexual violence committed during armed conflicts constitutes a severe human rights violation. In this regard, many of the more recent TJ processes have incorporated a gender-aware perspective in the development of their reparation policies. However, these normative-conceptual developments were not incorporated in the reparation process implemented after the dictatorship in Chile, or at least this evolution has gone unnoticed.

Since the Rettig Report—understandably—failed to take gender into account owing to its socio-historical context, it rendered invisible the experiences of women, which emerged and entered the official record nearly one decade later (Palestro, 2012). However, it is hard to comprehend why this approach did not inform measures implemented after the ratification of the international normative framework, which is the case of the Valech Report. Although Chapter II of this Report specifies the rights guaranteed and the manners in which they can be violated—which formed the basis of its results—it omits the categorization made in the Rome Statute of 1998 of a number of violent crimes of a sexual and gendered nature as crimes against humanity. It also omits UN Resolution no. 1325 of the year 2000, which addresses the impact of armed conflicts on women. In Chapter II, when referring to sexual violence against women, and despite citing the Rome Statute, the Report does not problematize the political and gender factors that grounded sexual violence; even more so, it stresses that “women were detained due to their ideas, their actions, and their political involvement, not for being women” (CNPPT, 2005, p. 252). This assertion leaves out the dimension of gender oppression, where the torturer State—as the utmost expression of the patriarchy crystallized in Chilean society as a whole—is the one subjecting, stigmatizing, and attacking women’s bodies to punish them for their political activism and instruct them in the fulfillment of their traditional role.

Maybe women—as pointed out in the Report—were not detained for their status as such, but it is undeniable that the specific mechanisms of torture, aimed at them and other feminized subjects, were grounded in it. The topic of sexual violence was not explicitly investigated by the Valech Commission, but it emerged as a spontaneous category in the statements received (CNPPT, 2005). We agree with Hiner that the sections that focus on women’s situation are still marked by “a limited command of the theoretical model of sexual and gendered violence. The approach has yet to overcome the binary oppositions based on biological sex and compulsory heterosexuality” (Hiner, 2009, p. 66). In Chapter VII, which profiles the victims, the section devoted to violence against women starts with an acknowledgment of violence against women, as stipulated in a number of international instruments. However, these references are only followed by a presentation of descriptive statistical data whose sociopolitical connotations are not discussed.

The report ends with a set of reparation proposals of three types: (1) institutional measures, (2) symbolic and collective measures, and (3) individual reparation measures, both legal and economic (CNPPT, 2005). Among the latter, health care measures are especially relevant, since they guarantee free-of-charge and preferential treatment through the Reparation and Integral Health Care Program (PRAIS). Furthermore, victims who suffered permanent physical consequences due to their imprisonment and torture are guaranteed to receive technical support and physical rehabilitation.

Nevertheless, two questions are worth asking about these guarantees: Are they consistent with the right to reparation to which the victims of human rights violations are entitled? Did these guarantees incorporate a gender-aware approach? After a rigorous analysis informed by a human rights approach, the answer is “no.” Reparations must have a transformational effect, that is, they must address not only the consequences of human rights violations, but also their underlying causes, granting victims continuing protection (UN, 2016). These requirements are analyzed below:

1. **Reparation of consequences**. A large body of literature indicates that the reparation process in Chile has not been integral (Bustamante-Danilo & Carreño-Calderón, 2020; Collins, 2019; Lira, 1996, 2011; Lira & Loveman, 2005). For decades, victims have demanded justice, which is a social determinant of their health. Indeed, its counterpart, impunity, is a recurrent topic in the therapeutic processes of victims treated through the PRAIS program, who identify it as a barrier to their recovery process (Cubillos Vega, 2023). This impunity becomes unbearable, for instance, when victims must endure re-traumatizing situations such as meeting their torturer(s) in public places. Furthermore, apart from access to justice, sexual violence victims also need specialized care and support in legal procedures in order to prevent new trauma and re-victimization. These services are not provided by PRAIS, nor are they part of the courts’ usual operations. Thus, the system fails to consider the fact that having to re-live their experience by making statements and facing their torturer places victims in a complex situation that requires an intersectoral approach. Another major deficit of the PRAIS program is the lack of systematization of user data (INDH, 2019), which makes it impossible to perform detailed analyses of the types of violence suffered and their health consequences. This is certainly needed if we consider that this population includes a large proportion of women (direct victims, widows, daughters, and mothers). Lastly, regarding economic reparation measures, the Single Reparation Benefit for victims acknowledged as such by the Valech Commission and surviving spouses can only be inherited by widows (women). However, it is worth asking, were only heterosexual men tortured?
2. **Response to underlying causes**. Reparation policies in Chile have overlooked what Cárdenas (2020) defines as the institutional and structural reforms that every political transition process should introduce to construct and reinforce a plural democracy and that, in the specific case of Chile, was necessary for addressing the historical inequalities and violences that the dictatorship exacerbated. Despite calls from the UN’s Office of the High Commissioner for Human Rights to transform society by addressing severe violations of economic, social, and cultural rights (ESCR), and despite regressions in terms of equality due to political violence, ESCRs remain excluded from TJ processes in Chile (Collins, 2019).
  With respect to sexual violence, the great difficulty of tackling the structural causes of gender-related crimes results from the naturalization of gender-related violence in society. In Chile, not enough structural or institutional reforms have been promoted in this regard, which has made it impossible to halt sexual violence crimes—both in the domestic sphere and within the context of public demonstrations—and gender-related torture. These human rights violations are not recognized as such by the State, causing them to go unpunished and allowing them to continue unabated. As the National Institute of Human Rights (INDH) has concluded (2019), violence against women is far from being eradicated; in fact, there were 58 femicides in 2020 (Red Chilena contra la Violencia hacia las Mujeres, 2021).
3. **Continuing protection**. This category must incorporate the following: (a) guarantees for beneficiaries to access and use the services offered and (b) the prevention of future rights violations.
  - Regarding access and use, the Human Rights Program of the Human Rights Agency (Subsecretaría de Derechos Humanos) excludes survivors, as its only duty is to collaborate with and process lawsuits filed in cases of political execution and disappearance, which means that it is not ensuring that all torture survivors can access the services offered.
  - With respect to prevention, it should be noted that symbolic and collective reparation measures included, among other efforts, human rights education, covering a number of topics related to citizenship education and values; however, this initiative left out gender-related violence in society and failed to encourage a critical discussion of other social issues derived from structural violence and discrimination.

Lastly, we must consider an issue that illustrates gender disparities in TJ policy itself, not only in Chile but also globally, and which results in the disproportionate representation of women when the categories “direct victims” and “indirect victims” are established. Analyzing Colombia’s TJ policy from a gender-aware perspective, Lyons (2011) warned that this disparity occurs because most of the victims who seek help through the TJ system do so in response to violence against others who are now absent. In Chile, gender disparity has been historically present but operating in the opposite direction, since public international law gradually evolved to address torture, which mostly affected men; therefore, this phenomenon has yet to be thoroughly analyzed from a broad gender-aware perspective, nor have the effects of structural discrimination been considered either (UN, 2016). For instance, in Chile, the rate of lawsuits initiated by women survivors of imprisonment and torture which have resulted in a sentence (33% of the total actions) surpasses the rate of women acknowledged as victims by the CNPPT (12.5% of the total cases), which for Collins (2019) suggests that women are more prone to take legal action due to the particularities of the violence that they have undergone. This situation highlights the need to study this phenomenon beyond its statistical manifestations. Lastly, it should be noted that if women’s victimization has been surprisingly invisibilized, the invisibilization has been dramatic for sexual or identity minorities.

## Gender-Based Torture within the Context of the 18-O Protests: The Failure of Non-repetition Assurances?

Torture as a method of gender-based violence was institutionally legitimized and became normal during the dictatorship and has remained crystallized until today, resurfacing during periods of social conflict in which human rights have been scarcely guaranteed (Jamett, 2012). This sexual violence has manifested itself, for instance, during the demonstrations in favor of free-of-charge and quality education of 2011, and more recently during the 18-O “social explosion.”

Gender-based violence, manifested through sexual abuses and violence, is evident in the acts, gestures, and vocabulary of the police (Carabineros), who exert very aggressive repression in order to achieve—like during the dictatorship—subordination, disciplining, and punishing, both physically and psychologically, through humiliation and control (Jamett, 2012).

The aim of these measures is to leave marks that remind victims that disobedience and autonomy are paid with pain and are regulated by the guilt and shame that eventually makes women feel responsible for the violence against them (Jamett, 2012, p. 93).

The present gender-based violence, in the form of gender-based torture, has manifested itself through the sexual violence exerted by State agents within the context of the demonstrations that began on October 18, 2019, with flagrant human rights violations occurring throughout the country until March 2020. Although they persist, these protests have abated due to the COVID-19 pandemic.

Within the context of 18-O, of all the lawsuits and events recorded by the National Institute of Human Rights (INDH, 2020), 93% were against the police, with 10.6% concerning acts of torture such as sexual violence. According to this source, 18-O human rights violations occurred in various places, but most took place in streets, police stations, and police vehicles. In total, 432 reports of human rights violations concerned acts if sexual violence. Of the 2825 victims, 563 were adult women, while 467 were children and adolescents. The INDH (2020) tallies reports (4074), victims (2835), and lawsuits (2349) separately. However, it is also worth noting that several cases of sexual violence are not reported due to victims’ fear of prejudice, stigmatization, and secondary victimization (INDH, 2019). The preliminary report of the INDH for the last quarter of 2019 stresses that sexual violence affected both men and women, but especially the latter. Forcible stripping was one of the most repeated practices, also including—in most cases—push-ups and cavity searches (anus and/or vagina) (INDH, 2019). These methods—which predominantly affected minors—are regarded as severe rights violations by the INDH, since they affect people’s psychological integrity and dignity by putting them in a vulnerable situation: first, they increase fears of potential mistreatment or abuse; second, when complemented by taunts and threats, they increase humiliation, thus constituting a type of torture.

In this regard, it is worth highlighting that five lawsuits were initiated due to sexual violations against males, which are considered especially severe due to the victims’ status as minorities prone to discrimination: two homosexual men and an immigrant man. Indeed, it was reported that, even though “forcible stripping, inappropriate touching, and rape threats affected a larger proportion of women, all the cases of sexual violation had male victims” (INDH, 2019, p. 48). Overall, insults with sexual connotations, LGTBI phobic taunts, threats of sexual torture, and blows to the genitals affected both men and women similarly.

Our review of two relevant episodes of human rights violations of our recent history (the civic-military dictatorship of 1973–1990 and 18-O) enables us to compare the characteristics and evolution of these crimes. Furthermore, it enables us to evaluate the effectiveness of the assurances of non-repetition included in post-dictatorship reparation policy.

Although the brutal gender-based torture methods of the dictatorship are not comparable to the types of sexual violence reported nowadays, the conceptual and normative classification of torture still applies to the latter. To exemplify the magnitude of the sexual violence reported in Chile, we could compare the number of cases of sexual violence documented in both periods, considering the total number of human rights violations acknowledged to have happened during the dictatorship (40,000) and the total number of reported cases within the context of the 18-O demonstrations until March 2020 (4075). Despite the large difference between the total number of human rights violations in both periods, there is a smaller gap between rates of sexual violence as a form of torture: cases of sexual violence during the dictatorship constitute 8% of the total, while those that occurred within the context of the 18-O demonstrations constitute 10% of the total. It is worth stressing that this comparison and the figures obtained are in no way an indicator of similarity. On the contrary, considering that the dictatorship lasted 17 years and the 18-O data were collected in only 6 months, the greater prevalence of this type of violence nowadays is clear.

The proportion of these types of crimes relative to the total number of human rights violations reported within the context of the 18-O demonstrations illustrates not only the crystallization of institutional gender-based violence, exerted by the State and its police force, but its expansion, which hints at the failure of the non-repetition assurances included in Chilean reparation policy. Furthermore, as noted in the previous section, reparation policy is marked by the absence of a specific gender-aware approach, which has certainly had an impact on this issue.

## Key Implications and Contributions

The Valech Report horrified Chilean society leading to the “official” acknowledgment of the sexual violence cases confirmed (although sexual violence cases were known long before the Report). Unfortunately, this knowledge did not eradicate gendered violence torture in the present.

On the contrary, as in so many other spheres of life, gender-based violence (against girls, heterosexual women, lesbians, gays, bisexuals, transgender people, ethnic minorities, or gender-nonconforming men and boys, in other words, against any female signifier) was omitted from and rendered invisible in reparation policies and non-repetition measures.

The fulfillment of the objective that guided this analysis has not been free from limitations. For instance, there is a variety of statistical sources which provide different data about the phenomena studied. Therefore, we chose to employ those issued by the CNPPT (which are not exact either, leading us to estimate that 3300 women were subjected to sexual violence) and the INDH (which are not disaggregated by sex or victimized community after January 2020). In addition, it should be noted that the two contexts studied are materially different: during the dictatorship, in the absence of the rule of law, silence was encouraged, probably leading to underrepresented sexual violence figures. In contrast, formal crime reporting methods exist nowadays. Nevertheless, it is also true that distrust of the police and the legal system, along with fears of secondary victimization, can result in an unwillingness to report crimes.

Regarding the contributions made by this study, it enabled us to establish a narrative that starts by outlining the gender-based violence suffered by the women imprisoned and tortured for political reasons during the dictatorship, followed by an analysis of the reparation policies implemented in response to these crimes against humanity from a perspective informed by human rights and gender principles, and closed by the identification of a number of biases that impact on the gendered violence exerted by State agents nowadays, as shown by the events reported within the context of the 18-O protests.

In particular, the analysis of reparation policies for human rights violations is relevant for our profession, given that social workers are part of the interdisciplinary work teams in the implementation of these policies (and sometimes even in their design). As Sánchez, (1990) points out, in Chile, social workers approached the task of defending human rights as a necessary and improvised response to the dictatorship. Along this path, they began to intervene professionally in this field based on an ethical commitment to our professional values of respect for human dignity, justice, and freedom (Sánchez, 1990; Cubillos-Vega, 2019). This intervention was initially welfare-based and supportive. However, soon extended to the popular organization of groups and communities so that they could become socio-political actors and denounce human rights violations, such as, for example, the existence of enforced disappeared persons (Sánchez,, 1990; Eroles, 1997). After the end of the dictatorship, the strategies described above continued to be used, adapting them to other contexts where rights violations occurred, as well as in detecting and dealing with gaps in existing social policies (Rubilar-Donoso, 2018; Cubillos Vega, 2023). The inclusion of the human rights approach in social policies has brought new challenges and requirements for social workers, who have had to incorporate citizenship perspectives and rights recognition practices into their professional and research repertoires (Rubilar-Donoso, 2018).

Our analysis of reparation measures—which rested on the premise that, according to international human rights law, reparations must have a transformational effect, addressing both the consequences of human rights violations and their underlying causes while also granting victims continuing protection—revealed serious shortcomings. Within the sphere of the reparation of consequences, no measures have been taken to prevent re-traumatization and re-victimization among torture victims. During legal processes, this bias has a specifically heavy impact on those affected by sexual violence, since there persists a tendency to stigmatize and blame the victim for the abuses suffered. Furthermore, the invisibilization of women and minorities in the systematization of statistical data constitutes another relevant element of bias in this field. This situation has made it impossible to conduct analyses aimed at evaluating their status as a result of the reparation measures adopted. With respect to reparation measures targeting the underlying causes of human rights violations, Chile has failed to implement the institutional and structural reforms needed to establish a plural democracy by reducing the social inequalities and structural violence heightened by the dictatorship (though historically present in our society). Deep-seated gender-based violence in Chilean society is the main cause of gendered torture, with failure to address it manifesting itself through the State violence that persists in society even at a higher rate than in the past, as illustrated in this article.

Lastly, regarding continuing protection and prevention, and in line with the conjecture advanced at the start of this article, assurances of non-repetition have been absent from TJ policy. Human rights education, across all sectors and levels, is one of the main tools for preventing human rights violations. That is, it should be aimed at both civil society and State agents as well as at the preschool, primary, secondary, and higher education levels. In the latter field, it should focus on the education of professionals committed to respecting, defending, and promoting human rights, as is the case of social workers. While leaving out the analysis of gender-based violence as structural violence, society and—certainly—State institutions will continue to be marked by the naturalization of violence against women and minimizing its multiple expressions and consequences.

The analysis conducted indicates that it is essential to take full advantage of the available instruments against torture and gender-based violence as a crime against humanity to prevent the impunity currently associated with these types of crimes and overcome the barriers that keep victims from accessing and benefiting from reparation policies.

Special care must be taken to avoid the secondary victimization that people experience when they need to give proof, since the process to collect the evidence tends to be discriminatory, or when they need to testify, as they are likely to be humiliated by their torturers. This doubtlessly requires the adoption of a gender-aware approach common to all the policies aimed at increasing victims’ well-being.

It is fundamental for the generalization of a gender-aware approach to be complemented with intersectoral reparation policies. In addition, reparation policies, apart from being intersectoral and informed by a gender-aware approach, must be truly comprehensive, addressing victims’ physical, psychological, and social well-being. In this regard, the cultural dimension has strategic importance: no matter how many economic, political, legal, or social welfare improvements are introduced, as long as Chilean society fails to establish a culture of respect for human rights, with a critical approach—not only based on normative components, but appreciating its political potential—violence in general, and specifically gender-based violence, will always be latent.

## Endnotes

^1^Even though the “victim” category has been challenged from certain feminist camps because it diminishes women’s agency, we use this term in this study to highlight that the acts of gendered torture to which they were subjected constitute crimes against humanity, committed by State agents, and are thus considered to be grave violations of international human rights law. In this context, victims are “persons who individually or collectively suffered harm, including physical or mental injury, emotional suffering, economic loss or substantial impairment of their fundamental rights, through acts or omissions that constitute gross violations of international human rights law, or serious violations of international humanitarian law.” (UN, 2005, art. 8). This category also gives rise to the right to take legal action and compels the State to provide reparations.

^2^Even though gender-based crimes tend to affect women and girls, they also target gay, transgender people, and gender nonconforming people, as well as men and boys, against whom it is exerted due to their failure to comply with socially assigned roles.

^3^The term *gender* conflates biological sex, gender identity, and sexual orientation, since repression and political violence, and specifically torture, are based on the application of binary and heteronormative gender norms which are deeply rooted in patriarchal culture.

^4^The Istanbul Protocol is a forensic evaluation instrument—a manual—for studying and documenting, in the medical, psychological, and legal fields, the sequelae exhibited by victims of torture and cruel, inhumane, and degrading treatment.

^5^In Chile, 1132 facilities were found to have been used as detention centers. Here, captors used torture methods such as electric shocks, repeated beatings, bodily injuries, hangings, forced positions, sexual violence, threats that relatives would be killed or tortured, simulated executions by firing squad, forcible stripping, humiliation and abuse, asphyxiation, exposure to extreme temperatures, seeing other detainees being tortured or executed, Russian roulette, confinement in inhuman conditions, and food and sleep deprivation (CNPPT, 2005; Hopenhayn, 2018).

^6^When discussing torture and gender, at least in the Chilean case, we cannot overlook the involvement of the women who collaborated with the torturer State. For Bunster (1991), their involvement can also be regarded as psychic torture, since they had a part in their own destruction, that of their family, and that of their community by acting as subordinates, with supplementary roles, and always under the command of military men.

^7^Among others, the *Program for Reparation and Integral Heath Care [and Human Rights]* (PRAIS), implemented in 1991, the measures outlined in Law no. 19123 (1992), which establishes a reparation pension and other benefits, or the *Discussion Table* of 2011.

^8^At the beginning of the democratic regime, in 1990, the Rettig Report only included the cases regarded as the most severe human rights violations, that is, those which resulted in presumed or certain death: people who were executed/assassinated or detained and made to disappear. In consequence, the reparation policies derived from the recommendations made in this report acknowledged these people as the sole victims, excluding torture survivors not only from reparations, but also from the official truth that this report institutionalized.

^9^Countries that have incorporated gender-related human rights abuses in their Truth commissions include Sierra Leone, South Africa, Peru, and East Timor.

^10^According to the stipulations of the 1993 World Human Rights Conference and the Beijing Platform for Action, adopted at the 4th World Conference on Women (1995), with respect to investigations of crimes against women in armed conflicts. In addition, the text notes the Chilean State’s agreements signed in the region (Convention of Belém do Pará).

^11^The data about the types of violence exerted between October 2019 and March 2020 are not disaggregated by sex on the INDH website (INDH, 2020). For data disaggregated by sex for the October–December 2019 period, see INDH (2019).

^12^The reports published by the CNVR and later by the CNPPT and the Presidential Advisory Commission for the Qualification of Disappeared Detainees, Prisoners Executed for Political Reasons, and Victims of Political Imprisonment and Torture (CPA) documented 40,000 cases of human rights violations affecting people detained and subsequently made to disappear, prisoners executed for political reasons, and victims of political imprisonment and torture between September 11, 1973, and March 11, 1990.

^13^“The CNPPT received the testimony of 3,399 women, who represented 12.5% of the participants (…) Nearly all the women reported having been affected by sexual violence, regardless of age, and 316 said they had been raped” (CNPPT, 2005, p. 291).

## References

[CR1] Androff D (2010). Truth and reconciliation commissions (TRCs): An international human rights intervention and its connection to social work. The British Journal of Social Work.

[CR2] Ardohain, V. (2014). Nuevos andares del Trabajo Social: el ejercicio del trabajador social en el acompañamiento a testigos-victimas del terrorismo de Estado. *X Jornadas de investigación, docencia, extensión y ejercicio profesional: "Transformaciones sociales, políticas públicas y conflictos emergentes en la sociedad argentina contemporánea*. http://sedici.unlp.edu.ar/handle/10915/43854

[CR3] Bermúdez, A. (2019, October 25). Protestas en Chile: "La tortura, los malos tratos en comisarías y la violencia con connotación sexual son preocupantes". BBC News Mundo. Retrieved April 15, 2022, from https://www.bbc.com/mundo/noticias-america-latina-50178678

[CR4] Bilbao, B. (2011). Violencia de género en los juicios del pasado y del presente. *Question*, 31 (1), 1–8. Retrieved March 23, 2022, from http://sedici.unlp.edu.ar/handle/10915/34475

[CR5] Buckley-Zistel, S., Beck, T. K., Braun, C., & Mieth, F. (2014). Transitional justice theories: An introduction. In *Transitional justice theories* (pp. 1–16). Routledge.

[CR6] Bunster, X. (1991). Sobreviviendo más allá del miedo. In X. Bunster, C. Enloe & R. Rodríguez (Eds.), *La mujer ausente: Derechos humanos en el mundo*, (pp. 41–62). Santiago: ISIS Internacional.

[CR7] Bustamante-Danilo, J. & Carreño-Calderón, A. (2020). Reparación simbólica, trauma y victimización: la respuesta del Estado chileno a las violaciones de derechos humanos (1973–1990). *Iconos Revista de Ciencias Sociales*, XXI (67), 39–59. 10.17141/iconos.67.2020.4231

[CR8] Camargo, K., Yáñez, S. & Chaparro, L. (2011). *Crímenes de lesa humanidad, violencia sexual y justicia de género en Colombia*. Bogotá: Corporación Sisma Mujer.

[CR9] Cárdenas M, Faúndez X, Hatibovic F, Villanueva J (2020). Posibilidades y Límites de la Reconciliación Política en Chile. Aproximaciones teóricas y conceptuales en estudios sobre cultura política, memoria y derechos humanos.

[CR10] Castañeda P, Salame A (2019). Memoria profesional y Trabajo Social chileno. Derechos humanos y dictadura cívico militar. Revista Katálysis.

[CR11] Cerruti, P. (2017). La problematización de la violencia de género como práctica del terrorismo de Estado en la Argentina: Consideraciones genealógicas y análisis de sus modos recientes de formalización jurídica. *e-l@tina Revista electrónica de estudios latinoamericanos*, 15(58), 1–15.

[CR12] CNPPT, Comisión Nacional sobre Prisión Política y Tortura. (2005). Informe de la Comisión Nacional sobre Prisión Política y Tortura (Valech I). Retrieved March 19, 2022, from https://bibliotecadigital.indh.cl/handle/123456789/455

[CR13] Collins C, Vargas F (2019). La memoria en los tiempos del cólera: Verdad, justicia, reparaciones, y garantías de no repetición por los crímenes de la dictadura chilena. Informe anual sobre derechos humanos en chile 2019.

[CR14] Cubillos-Vega, C. (2019). Bienestar social: un objetivo compartido. Sobre la alianza entre los derechos humanos y el trabajo social. *Arbor, 195*(791), a493. 10.3989/arbor.2019.791n1006

[CR15] Cubillos Vega, C. (2023). La reparación del trauma social por crímenes de lesa humanidad en Chile: el caso del Programa de Reparación y Atención Integral de Salud analizado bajo el Enfoque de derechos humanos. *Alternativas. Cuadernos de Trabajo Social, 30*(1), 1–28. 10.14198/ALTERN.21338

[CR16] Denzin N, Lincoln Y, Denzin N, Lincoln Y (1994). Introduction: Entering the field of qualitative research. Handbook of Qualitative Research.

[CR17] Eroles, C. (1997). Derechos humanos: compromiso ético del trabajo social. In C. Eroles (comp), *Los Derechos Humanos. Compromiso ético del trabajo social* (pp. 13–64). Buenos Aires: Espacio editorial.

[CR18] Gil de Camín, M. (1997). Trabajo Social y Derechos Humanos: una experiencia de campo en Cuyo. In C. Eroles (comp), *Los Derechos Humanos. Compromiso ético del trabajo social* (pp. 91–115). Buenos Aires: Espacio editorial.

[CR19] González A (2014). Violencias de género constitutivas de crímenes de lesa humanidad y genocidio. El Caso De Guatemala. Aletheia.

[CR20] Hayner P (2001). Unspeakable Truths: Facing the Challenge of Truth Commissions.

[CR21] Healy L (2008). Exploring the history of social work as a human rights profession. International Social Work.

[CR22] Hiner H (2009). Voces soterradas, violencias ignoradas: Discurso, violencia política y género en los Informes Rettig y Valech. Latin American Research Review.

[CR23] Hiner H (2015). “Fue bonita la solidaridad entre mujeres”: Género, resistencia, y prisión política en Chile durante la dictadura. Revista Estudos Feministas.

[CR24] Hopenhayn, D. (2018). *Así se torturó en Chile (1973–1990). Relatos del Informe Valech*. Santiago: La Copa Rota.

[CR25] Hourcade, S., Ghelfi, F., Palmás, L., & Perelman, M. (2018). *Comisiones de la Verdad de Chile: Verdad y Reparaciones como Política de Estado*. Bergen: Christian Michelsen Institut, CMI.

[CR26] Ife J (2001). Local and global practice: Relocating social work as a human rights profession in the new global order. European Journal of Social Work.

[CR27] INDH, Instituto Nacional de Derechos Humanos. (2019). *Informe Anual Situación de los Derechos Humanos en Chile 2019*. Santiago: Instituto Nacional de Derechos Humanos. Retrieved March 12, 2021, from https://bibliotecadigital.indh.cl/bitstream/handle/123456789/1701/Informe%20Final-2019.pdf?sequence=1&isAllowed=y

[CR28] Jamett, F. (2012). Duerme tranquila niña inocente: Violencia sexual policial contra niñas y mujeres jóvenes en las manifestaciones del movimiento estudiantil el año 2011. In E. Águila (Ed.), *Mujeres y violencia: Silencios y resistencias* (pp. 84–95). Santiago: Red Chilena contra la Violencia Doméstica y Sexual.

[CR29] Jara D, Badilla M, Figueiredo A, Cornejo M, Riveros V (2018). Tracing Mapuche exclusion from post-dictatorial truth commissions in Chile: Official and grassroots initiatives. International Journal of Transitional Justice.

[CR30] Joinet, L. (1997). *The administration of justice and the human rights of detainees. Revised final report on the question of the impunity of perpetrators of human rights violations (civil and political)*. E/CN.4/Sub.2/1997/20. Economic and Social Council. United Nations. Retrieved February 03, 2022, from https://documents-dds-ny.un.org/doc/UNDOC/GEN/G97/141/42/PDF/G9714142.pdf?OpenElement

[CR31] Ley 19123. (1992). Crea Corporación Nacional de Reparación y Reconciliación, establece pensión de reparación y otorga otros beneficios en favor de personas que señala. Biblioteca del Congreso Nacional de Chile. Retrieved March 18, 2022, from https://bcn.cl/2o4yq

[CR32] Lira, E. (1996). El legado de las violaciones de derechos humanos y la transición política. In E. Lira & I. Piper (Eds.), *Reparación, Derechos Humanos y Salud Mental* (pp. 11–20). Santiago: ILAS & Ediciones ChileAmérica CESOC.

[CR33] Lira, E. (2011). Verdad, reparación y justicia: El pasado que sigue vivo en el presente. In IIDH (Ed.), *Contribución de las políticas de verdad, justicia y reparación a las democracias en América Latina* (pp. 86–127). San José de Costa Rica: Instituto Interamericano de Derechos Humanos.

[CR34] Lira, E. (2017). Mujeres embarazadas víctimas de tortura: las denuncias a la Comisión Nacional sobre Prisión Política y Tortura. In K. Bilbija, A. Forcinito & B. Llanos (eds.), *Poner el cuerpo: Rescatar y visibilizar las marcas sexuales y de género de los archivos dictatoriales del Cono Sur* (pp. 107–129). Santiago: Cuarto Propio.

[CR35] Lira, E. & Loveman, B. (2005). *Políticas de Reparación en Chile 1990–2004*. Santiago: LOM.

[CR36] Lyons, A. (2011). Reconocer la discriminación de género: La igualdad como un requisito para el desarrollo de políticas de justicia transicional legítimas y eficaces. In M. Moreno (Ed.), *Políticas públicas que hacen justicia: cuatro temas en la agenda de reparación en Colombia* (pp.139–187). Bogotá: Centro Internacional para la Justicia Transicional (ICTJ).

[CR37] Maravall J (2008). Mujeres en movimiento: Las prisioneras políticas bajo la dictadura militar chilena (1973–1990). Cuestiones de género.

[CR38] Odio, E. (1997). Protección de los Derechos Humanos de las Mujeres. In G. Pacheco (Coord.), *Protección internacional de los derechos humanos de las mujeres* (pp. 25–38). San José de Costa Rica: Instituto Interamericano de Derechos Humanos.

[CR39] Olivera-Williams, M. R. (2017). Maldito cuerpo de mujer: violencia de género y violencia sexual dentro del terrorismo de estado en Argentina y Chile. In K. Bilbija, A. Forcinito, & B. Llanos (Eds.), *Poner el cuerpo: Rescatar y Visibilizar las Marcas Sexuales y de Género de los Archivos Dictatoriales del Cono sur* (pp. 61–83). Editorial Cuarto Propio.

[CR40] Palestro, S. (2012). Violencia sexual en la tortura contra mujeres: un silencio con historia. In E. Águila (Ed.), Mujeres y violencia: silencios y resistencias (pp. 78–83). Santiago: Red Chilena contra la Violencia Doméstica y Sexual.

[CR41] Pérez-Sales P, Zraly M (2018). From sexualized torture and gender-based torture to genderized torture: The urgent need for a conceptual evolution. Torture.

[CR42] Red Chilena contra la Violencia hacia las Mujeres. (2021). Registro de femicidios. Retrieved January 10, 2021, from http://www.nomasviolenciacontramujeres.cl/registro-de-femicidios/

[CR43] Rubilar-Donoso G (2018). Trabajo Social y Derechos Humanos: perspectivas, posibilidades y desafíos a partir de la experiencia chilena. Trabajo Social Global – Global Social Work.

[CR44] Sánchez, D. (1990). *Trabajo social y derechos humanos: Compromiso con la dignidad: La experiencia chilena*. Buenos Aires: Humanitas.

[CR45] Segato R (2003). Las estructuras elementales de la violencia: Ensayos sobre género entre la antropología, el psicoanálisis y los derechos humanos.

[CR46] Segato R (2016). La guerra contra las mujeres.

[CR47] Segato, R. (2020). Prólogo. In M. Lewin & O. Wornat (Eds.)*, Putas y guerrilleras* (pp. 12–17). Buenos Aires: Planeta.

[CR48] Tito, C. (2019, September 10). *Justicia y reparación para mujeres torturadas en dictadura: una deuda pendiente en Chile*. El Mostrador. Retrieved March 27, 2022, from https://www.elmostrador.cl/braga/2019/09/10/justicia-y-reparacion-para-mujeres-torturadas-en-dictadura-una-deuda-pendiente-en-chile/

[CR49] Tornay, L. & Alvarez, V. (2012). Tomar la palabra. Memoria y violencia de género durante el terrorismo de Estado. *Aletheia*, 2(4), 1–14.

[CR50] UN, United Nations. (2005). Basic principles and guidelines on the right to a remedy and reparation for victims of gross violations of international human rights law and serious violations of international humanitarian law. General Assembly resolution 60/147. Retrieved February 03, 2022, from https://www.ohchr.org/en/instruments-mechanisms/instruments/basic-principles-and-guidelines-right-remedy-and-reparation

[CR51] UN, United Nations. (2016). Report of the Special Rapporteur on torture and other cruel, inhuman or degrading treatment or punishment. General Assembly. A/HRC/31/57. Retrieved February 03, 2022, from https://documents-ddsny.un.org/doc/UNDOC/GEN/G16/000/97/PDF/G1600097.pdf?OpenElement

[CR52] Van Boven, T. (1993). *Estudio relativo al derecho de restitución, indemnización y rehabilitación a las víctimas de violaciones flagrantes de los derechos humanos y las libertades fundamentales*. Ginebra: Comisión de Derechos Humanos de Naciones Unidas.

